# Eosinophilic Duodenitis Presenting As Severe Iron Deficiency Anemia: A Rare Presentation

**DOI:** 10.7759/cureus.71595

**Published:** 2024-10-16

**Authors:** Rajeshwaree Bal, Nitish Batra, Sourya Acharya, Utkarsh Pradeep, Sunil Kumar

**Affiliations:** 1 Medicine, Jawaharlal Nehru Medical College, Datta Meghe Institute of Higher Education and Research, Wardha, IND

**Keywords:** anemia, duodenitis, eosinophilic, gastroenteritis, iron deficiency anemia

## Abstract

Eosinophilic gastrointestinal disorders (EGIDs) are representative of eosinophilic invasion of various sections of the gastrointestinal tract and do not pertain to any secondary causes. To affirm the diagnosis of eosinophilic infiltration of the gastrointestinal tract, tissue biopsy evidence must be demonstrated. Both iron deficiency anemia and gastritis symptoms may be brought on by mucosal eosinophilic infiltration. One of the most common dietary deficiencies globally is iron deficiency anemia which affects most developing populations. Reduced dietary iron and poor iron absorption are significant reasons for iron deficiency anemia (IDA), although the most common causes are menstruation and GI bleeding in women. Here, we describe a case of an 18-year-old female, with iron deficiency anemia, who was diagnosed with eosinophilic duodenitis.

## Introduction

Eosinophilic gastrointestinal disorders (EGIDs) are a rare and poorly understood gastrointestinal disorder characterized by eosinophilic infiltration of the gastrointestinal tract, often corresponding to symptoms such as abdominal discomfort, vomiting, dizziness, and irregular bowel movements. Since Kajiser initially reported eosinophilic gastroenteritis (EGE) in 1937, the literature has documented roughly 300 instances. According to estimates, there are 8.4 to 28 cases of it for every 100,000 persons in the US [1]. EGE is believed to be brought on by an aberrant immunological response to environmental or dietary antigens, which causes an influx of eosinophils into the intestinal wall [2]. Numerous factors, including genetics, age, allergy comorbidities, and allergen exposures, have an impact on the mechanisms underlying the development and progression of EGIDs [3].

One of the most common complications of EGE is iron deficiency anemia (IDA). 800,000 fatalities globally are attributed to IDA, a global public health issue that affects 7.21-13.96 persons per 1000 person-years [4]. IDA is a condition in which the body does not have enough iron to meet the metabolic and other requirements needed by the body. IDA is often caused by chronic bleeding or inadequate iron intake, but it can also be a consequence of malabsorption or impaired iron absorption. In patients with EGE, IDA may be due to chronic inflammation and mucosal damage in the gastrointestinal tract, leading to impaired iron absorption and increased iron loss.

Despite its relatively rare occurrence, EGE can have significant consequences for patients' quality of life and overall health. Furthermore, IDA can exacerbate symptoms of EGE, such as fatigue, weakness, and shortness of breath. Therefore, it is essential to recognize the relationship between EGE and IDA and to develop effective treatments for these conditions.

## Case presentation

An 18-year-old woman presented to the internal medicine department with complaints of shortness of breath on exertion, tingling sensation in bilateral lower limbs, and generalized weakness. A patient has no significant past medical history and has a regular menstruation history. On general examination, pallor, glossitis, and koilonychia were present. Per abdomen examination, it did not reveal any organomegaly. Other system examinations were normal. Investigations are shown in Table 1.

**Table 1:** Laboratory investigations done and their values found in patient. Hb: Hemoglobin, HCT: Hematocrit test, MCV: Mean corpuscular volume, MCHC: Mean corpuscular hemoglobin concentration, MCH: Mean corpuscular hemoglobin, TIBC: Total iron binding capacity, ANA: Antinuclear antibody, ESR: erythrocyte sedimentation rate.

Laboratory Investigations	Values	Biological reference range
Hb (g/dL)	7.6	13-15
HCT (%)	24.2	37-47
MCV (fL)	58	80-98
MCHC(g/dL)	31.2	33-36
MCH (pg)	19.5	28-32
Ferritin (ng/mL)	22	24-307
Iron (mcg/dL)	28	50-150
TIBC (mcg/dL)	475	250-310
Vit B12 (pg/mL)	296	200-800
Absolute Eosinophil Count	487	30-300
ANA	0.8	0.9-1.1
ESR	<20mm/hr (women under 50 years old)	16mm/hr
Stool guaiac test	Negative	
C-Reactive protein	Negative	

**Figure 1:**
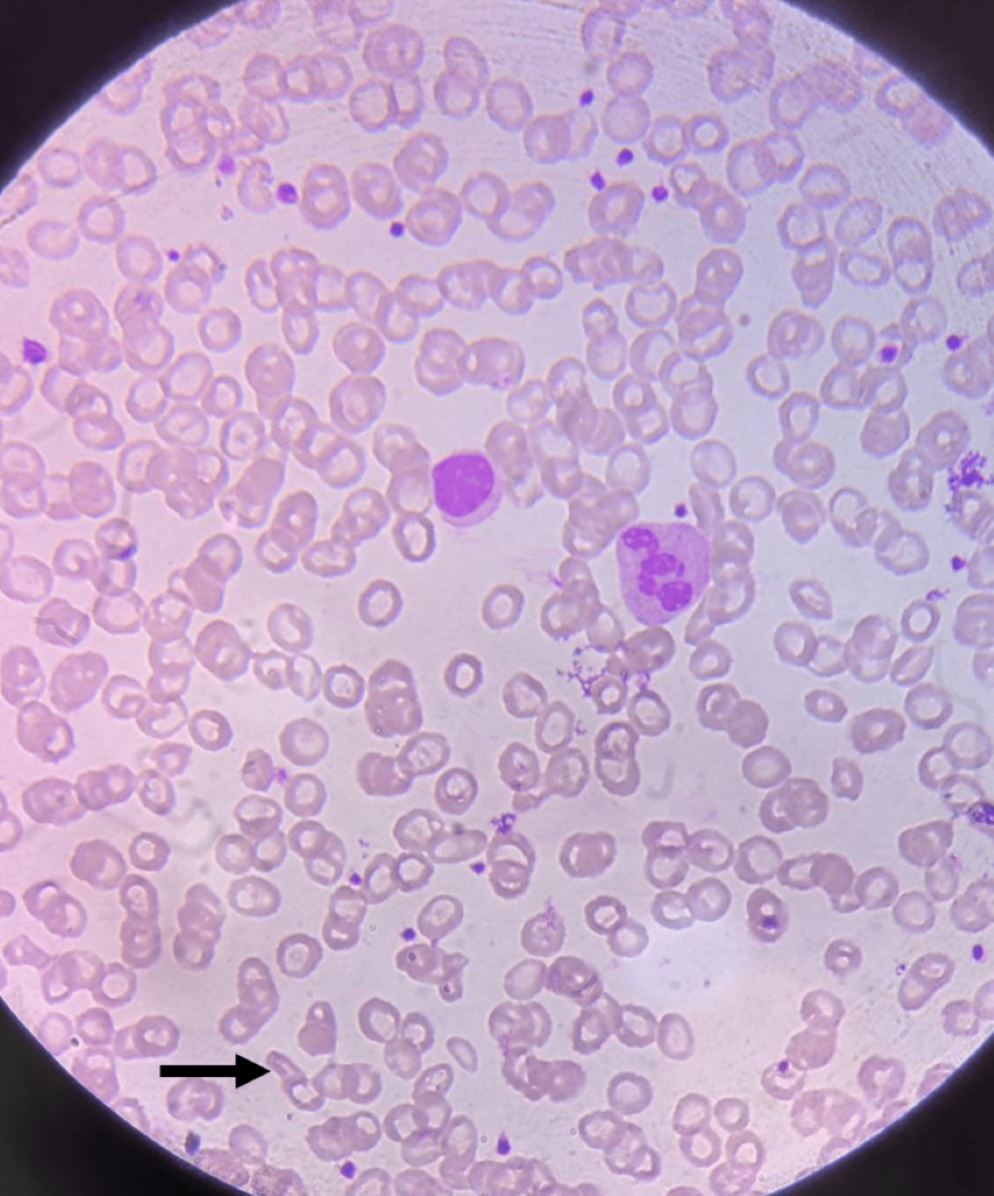
A peripheral smear slide with black arrow showing cigar/pencil shaped cells in microcytic hypochromic anemia.

Peripheral smear showed a picture of microcytic hypochromic anemia with pencil cells, as shown in Figure 1.

**Figure 2:**
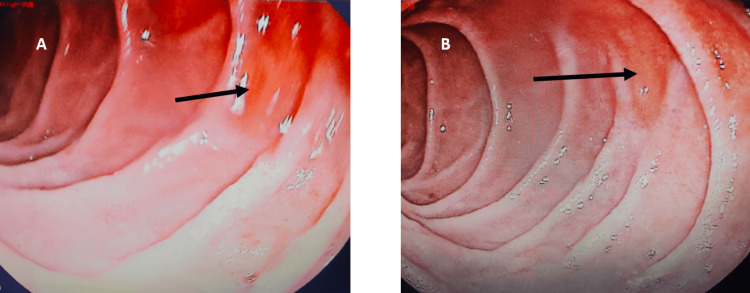
A&B shows an upper GI endoscopy with black arrow pointing towards erythematous patches in duodenum. GI: gastrointestinal

To evaluate the cause of iron deficiency anemia, the patient underwent gastroscopy, which revealed erythematous patches in the first and second part of the duodenum (Figure 2) and multiple biopsies were taken.

**Figure 3:**
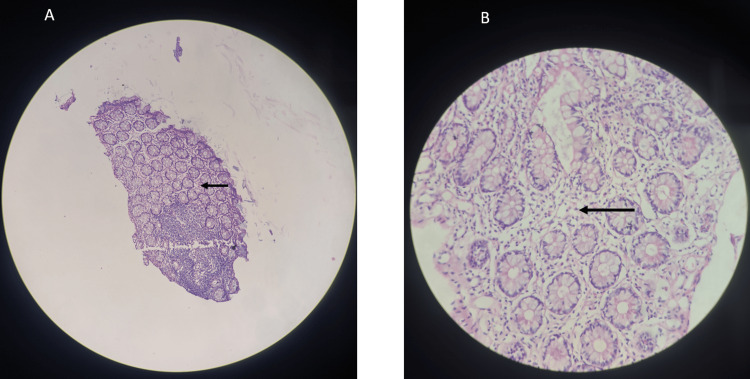
Hematoxylin and eosin stain of the duodenum The given section stained with hematoxylin and eosin stain (A-low power view 10X, eHigh power view 40X) shows areas of the duodenum with abundant eosinophils scattered in between cells suggestive of eosinophilic duodenitis. Black arrows highlights eosinophils.

Biopsy revealed eosinophilic duodenitis (EOD) (Figure 3).

After calculating the iron requirement via Ganzoni's formula [5], which came out to be 950 mg iron, she was started on the treatment of injecting iron sucrose 200 mg IV for six doses along with folic acid 5 mg per oral once a day. The patient was later started on prednisolone 1 mg/kg body weight. The patient was also advised, and the need for dietary restriction was explained. Her weakness had improved significantly, and associated complaints were resolved. She was instructed to follow up on an outpatient basis and repeat gastroscopy after six weeks.

## Discussion

A group of immune-mediated chronic disorders known as eosinophilic gastrointestinal diseases (EGIDs) are distinguished by eosinophil pathologic infiltration of the GI tract and clinically manifested as gastrointestinal symptoms. Based on the location of eosinophilic infiltration, EGIDs are categorized as eosinophilic duodenitis and eosinophilic gastritis. Although the exact origin of eosinophilic duodenitis is unknown, prior research has shown that people with this condition frequently also have concomitant allergic conditions such as food allergies, asthma, or peripheral eosinophilia [6].

When Jaffe et al. used flow cytometry on three patients with eosinophilic gastroenteritis, they observed increased IL-4 and IL-5 production, two cytokines closely linked to eosinophil activation and IgE synthesis, respectively [7]. The area of the GI tract affected and the tissue layer where eosinophils have invaded both affect how an EGID presents clinically. Typical non-specific symptoms of EGE include nausea, vomiting, diarrhea, abdominal pain, and weight loss. If the small intestine is involved, malabsorption can also be observed and can result in a variety of nutritional deficits [8]. 

Oral iron absorption by the gastrointestinal tract is inhibited in anemic patients due to the hepcidin response [5]. Our diagnosis is hinged on the fact that our patient despite nutritional supplements and a standard regimen treatment for iron deficiency treatment, did not show any response as well as the absence of any other pathological cause involving the gastrointestinal system or evidence of GI bleed.

Endoscopy and the discovery of many eosinophils in a single HPF on the histopathologic assessment of biopsies are the gold standards for diagnosis. The current threshold values for the diagnosis of eosinophilic duodenitis in the duodenum are >30 eosinophils per HPF in >3 HPF, according to the Food and Drug Administration (FDA). The cornerstone of treatment other than dietary restrictions is corticosteroids, to which the majority of patients respond symptomatically. In most cases, symptom alleviation happens a few weeks after treatment starts [9]. However, their toxicity limits their long-term usage [10].

## Conclusions

In addition to iron-deficiency anemia, protein-losing enteropathy, and malnourishment, mucosal eosinophilic infiltration can result in gastritis symptoms. Even blockage and perforation, as well as eosinophilic ascites or pleural effusion, can result from eosinophilic infiltration of the gut wall's deeper layers. Endoscopically acquired mucosal biopsies may fail to detect the diagnosis in such circumstances. A patient who presents with gastroenteritis should have eosinophilic gastritis taken into account in the broad differential diagnosis. It does, however, present a diagnostic issue. When mucosal biopsies taken by endoscopy are not diagnostic, full-thickness biopsies ought to be taken into consideration. Treatment alternatives like PPIs, steroids, leukotriene modifiers, and diet alterations cannot be used until the diagnosis has been made.
